# Resveratrol Alleviates the Prenatally Intermittent Hypoxia‐Induced Cognitive Impairment in Male Offspring Mice Through Modulating the SIRT1/HIF‐1α Pathway

**DOI:** 10.1002/brb3.70413

**Published:** 2025-03-14

**Authors:** Xun He, Ziwei Cao, Xinyi Chen, Jie Hu, Jiaxuan Li, Xinhui Jia, Juncang Wu, Xuechun Liu

**Affiliations:** ^1^ Department of Neurology Hefei Second People's Hospital Affiliated to Bengbu Medical University Hefei Anhui China; ^2^ Department of Neurology the Second People's Hospital of Hefei Hefei Anhui China; ^3^ Department of Neurology Hefei Hospital Affiliated to Anhui Medical University (the Second People's Hospital of Hefei) Hefei Anhui China

**Keywords:** cognitive impairment, prenatal intermittent hypoxia, resveratrol, SIRT1/HIF‐1α pathway

## Abstract

**Objective:**

Growing evidence indicate that prenatal intermittent hypoxia (PIH) exposure can have an impact on the critical brain nerve development of the fetus, resulting in cognitive deficits in the offspring mice. Resveratrol, recognized for its anti‐inflammatory and antioxidant capabilities, has potential to ameliorate synaptic dysfunction, which in turn may contribute to its positive influence on cognitive impairment. Nevertheless, the precise manner in which resveratrol mitigates cognitive deficits stemming from maternal hypoxia remains to be fully elucidated, including the specific mechanisms at play.

**Method:**

Pregnant C57BL/6J mice were exposed to intermittent hypoxia using the ProOx‐100 animal hypoxia control system during late pregnancy for 7 consecutive days. Resveratrol treatment at 40 mg/kg dosage was given to the subjects between postnatal Days 60 and 88. Morris water maze test was utilized to assess the cognitive capabilities of the male offspring mice. ELISA was employed to measure the concentrations of pro‐inflammatory cytokines within the hippocampal region of the mature offspring mice. The expression levels of the regulatory proteins SIRT1 and HIF‐1α, along with the synaptic plasticity markers SYP, Arc, GAP‐43, and PSD‐95 were measured by employing western blotting and RT‐qPCR.

**Results:**

In male offspring subjected to PIH, there was a marked decline in learning and memory capabilities, accompanied by increased levels of IL‐1, IL‐6, and TNF‐α within the hippocampal region. Administration of resveratrol notably ameliorated the cognitive deficits observed in these offspring and mitigated the heightened levels of pro‐inflammatory cytokines. Furthermore, exposure to PIH was associated with a reduction in the expression of key proteins such as SIRT1, HIF‐1α, Arc, GAP‐43, PSD‐95, and SYP within the hippocampal region, which were subsequently normalized following resveratrol intervention.

**Conclusion:**

The findings from our study indicate that resveratrol potently alleviates learning and memory impairments, the inflammatory response, and impairments in synaptic plasticity, which are induced by maternal intermittent hypoxia, by modulating the SIRT1/HIF‐1α signaling pathway.

## Graphical abstract

Prenatal intermittent hypoxia (PIH) exposure could provoke inflammation and synaptic dysfunction leading to cognitive impairment, resveratrol potentially reversing cognitive deficits by influencing the SIRT1/HIF‐1α pathway.

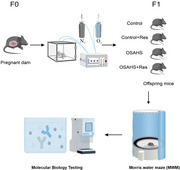

## 1 Introduction

Obstructive sleep apnea–hypopnea syndrome (OSAHS) is caused by recurrent collapse of the upper airway or central factors leading to intermittent nocturnal hypoxia and frequent awakenings. Due to changes in the endocrine system and anatomical structure related to pregnancy, particularly in the late stage of pregnancy, epidemiological studies have found that 26% of pregnant women will experience sleep apnea in the later stage of pregnancy (Wilson et al. 2022). The early stages of life represent a sensitive stage in the maturation process of the neural circuitry, with its optimal development being crucial for ensuring lifelong cognitive well‐being, as well as for the development of executive functions, attention, learning, and memory functions (B. Wang, Zeng, et al. 2021). Pregnancy‐related sleep apnea can lead to intermittent hypoxia in the uterus, which serves as a prenatal stress event that may affect fetal development. Extensive research has shown a close correlation between prenatal intermittent hypoxia and cognitive impairment in offspring (Sokolov et al. 2022). Furthermore, research has indicated that exposure to hypoxia during gestation is linked to detrimental effects on the development of the hippocampus in offspring mice, specifically within the region responsible for learning and memory, leading to adverse impacts on cognitive abilities (Zhuravin et al. 2019). Nevertheless, despite existing literature documenting the detrimental impacts of gestational hypoxia on the cognitive abilities of offspring, the precise molecular and biological processes at play remain elusive, and the need for further research is evident.

Silent information regulator 1 (SIRT1) stands as the most deeply investigated member of the sirtuin family, which encompasses SIRT1 through SIRT7. These sirtuin enzymes function as deacetylases reliant on nicotinamide adenine dinucleotide (NAD+), indicating their capacity to serve as receptors sensitive to the NAD+/NADH ratio. This sensitivity establishes a direct correlation between sirtuin function and the cell's metabolic and energetic conditions, thereby bridging the metabolic and energy status domains (X. Li and Kazgan 2011). SIRT1 is implicated in a spectrum of crucial physiological processes, such as the regulation of gene expression, cell death, energy preservation, lifespan extension, and the sustenance of cognitive health. Furthermore, SIRT1 exerts control over the stability and activity of a variety of transcription factors, including p53, FOXO, E2F1, NF‐κβ, PGC‐1α, and the hypoxia‐inducible factor (HIF) (Chen et al. 2020; Laemmle et al. 2012; Shin et al. 2021).

HIF serves as a pivotal regulator of oxygen balance under conditions of reduced oxygen availability, exerting significant influence on a variety of neurological disorders and cognitive impairments. The HIF dimer consists of oxygen‐sensitive active units (α‐subunits) and structural units (β‐subunits), with the stability and activity of HIF mainly determined by HIF‐1α (Semenza 2001). SIRT1 can bind to HIF‐1α and deacetylate at lysine 674, which is acetylated by p300/CBP‐related factors (PCAF), thereby inhibiting HIF‐1α through blocking the recruitment of p300 (Dong et al. 2016). Under hypoxic conditions, the NAD+/NADH ratio diminishes as a result of diminished NADH consumption in the mitochondria coupled with enhanced NADH generation via glycolysis. This shift results in reduced SIRT1 expression and the subsequent acetylation and activation of HIF‐1α (Lim et al. 2010). Studies have indicated that HIF‐1α contributes to cognitive deficits in septic mice by inducing mitochondrial dysfunction, neuronal cell death, and inflammatory processes (Zhao et al. 2022).

Recent studies have underscored the pivotal role of inflammatory responses and oxidative stress in the detrimental impacts of hypoxic conditions on cognitive capabilities (J. Liu et al. 2019). Notably, heightened concentrations of inflammatory cytokines, including interleukin‐1 (IL‐1), interleukin‐6 (IL‐6), and tumor necrosis factor‐α (TNF‐α), have been detected in the hippocampal region of offspring subjected to hypoxia. These cytokines are known to disrupt synaptic plasticity and neuronal function, thereby impairing learning and memory (Mac Giollabhui et al. 2020). In addition, oxidative stress resulting from hypoxic conditions can lead to neuronal damage and synaptic dysfunction, further exacerbating cognitive deficits.

The activity‐regulated cytoskeleton‐associated protein (Arc) is a product of an immediate‐early gene that dynamically translocate between dendritic and nuclear compartments. It is integral to the modulation of synaptic plasticity and the pruning of synapses (Epstein and Finkbeiner 2018). Evidence indicates that dysregulated Arc expression could disrupt synaptic plasticity, consequently impacting learning and memory processes (Mabb and Ehlers 2018). The postsynaptic density protein 95 (PSD‐95) is instrumental in preserving the structural integrity of synapses and enhancing synaptic plasticity (Murack et al. 2023). Synaptophysin (SYP) is implicated in the regulation of dendritic and axonal growth and differentiation, along with the release of neurotransmitters (L. Zhang et al. 2014). Both SYP and PSD‐95, serving as synaptic markers, are essential for sustaining cognitive function (Y. Liu et al. 2018). Growth‐associated protein 43 (GAP‐43), a presynaptic membrane protein, is predominantly found in the hippocampus and associated cortical areas, exhibiting heightened expression during neuronal maturation and the establishment of synaptic connections. This protein is involved in the regulation of synaptogenesis in the adult brain, synaptic plasticity, and axonal elongation. GAP‐43 is pivotal for modulating synaptic vesicle fusion and the process of synaptic transmission, thereby playing a significant role in synaptic function (De Moliner et al. 2015). Despite the growing body of evidence linking hypoxia exposure to cognitive impairments, there is a paucity of effective therapeutic interventions to mitigate these effects. Resveratrol (RES) is a polyphenolic compound primarily found in grape skin, mulberries, and common food sources such as peanuts. Research suggests that RES can act as an activator of SIRT1, exerting various biological activities including cancer prevention, anti‐inflammatory effects, improved circulation, anti‐aging effects on atherosclerosis, and inhibition of cell proliferation (C. Li et al. 2018). However, it is still unclear whether and how RES can participate in the mechanisms of offspring cognitive changes induced by intrauterine hypoxia.

In our research, we concentrated on scrutinizing the SIRT1/HIF‐1α signaling pathway, known for its involvement in the modulation of inflammatory responses, oxidative stress, and synaptic plasticity. The study aimed to delve into the underlying causes of cognitive decline in male C57BL/6J mice that experienced prenatal hypoxic conditions and to explore the potential of RES in mitigating these cognitive deficits. Specifically, we examined whether RES's beneficial impact, if any, is mediated through the modulation of pro‐inflammatory cytokines and proteins associated with synaptic plasticity within the hippocampal areas of the hypoxia‐exposed progeny of C57BL/6J mice.

## 2 Methods and Materials

### 2.1 Experimental Animals

The C57BL/6J mice were obtained from Beijing Vital River Laboratory Animal Technology Corporation. These animals were provided with unrestricted access to both food and water during an acclimatization period of 1 week. The environmental conditions were carefully controlled, with a temperature maintained at (22 ± 1)°C and relative humidity at (50 t 5)%. The lighting regimen was set to a 12‐h cycle alternating between light and darkness. For mating, male and female C57BL/6J mice were cohoused in a 1:2 (male to female) ratio within their enclosures starting at 9:00 p.m. The following morning at 7:00 a.m., the presence of a vaginal plug was verified to confirm mating, marking Day 0 of gestation (G0). Following the detection of a mating plug, the pregnant females were then isolated for individual housing. The mating process was facilitated in a 1:2 ratio, and the confirmation of a mating plug the next day led to the separation of pregnant females (R. Wei et al. 2023). The research was executed under the sanction of the Animal Experimentation Committee affiliated with the Anhui Medical University's Hefei Hospital.

### 2.2 Treatments

**FIGURE 1:**
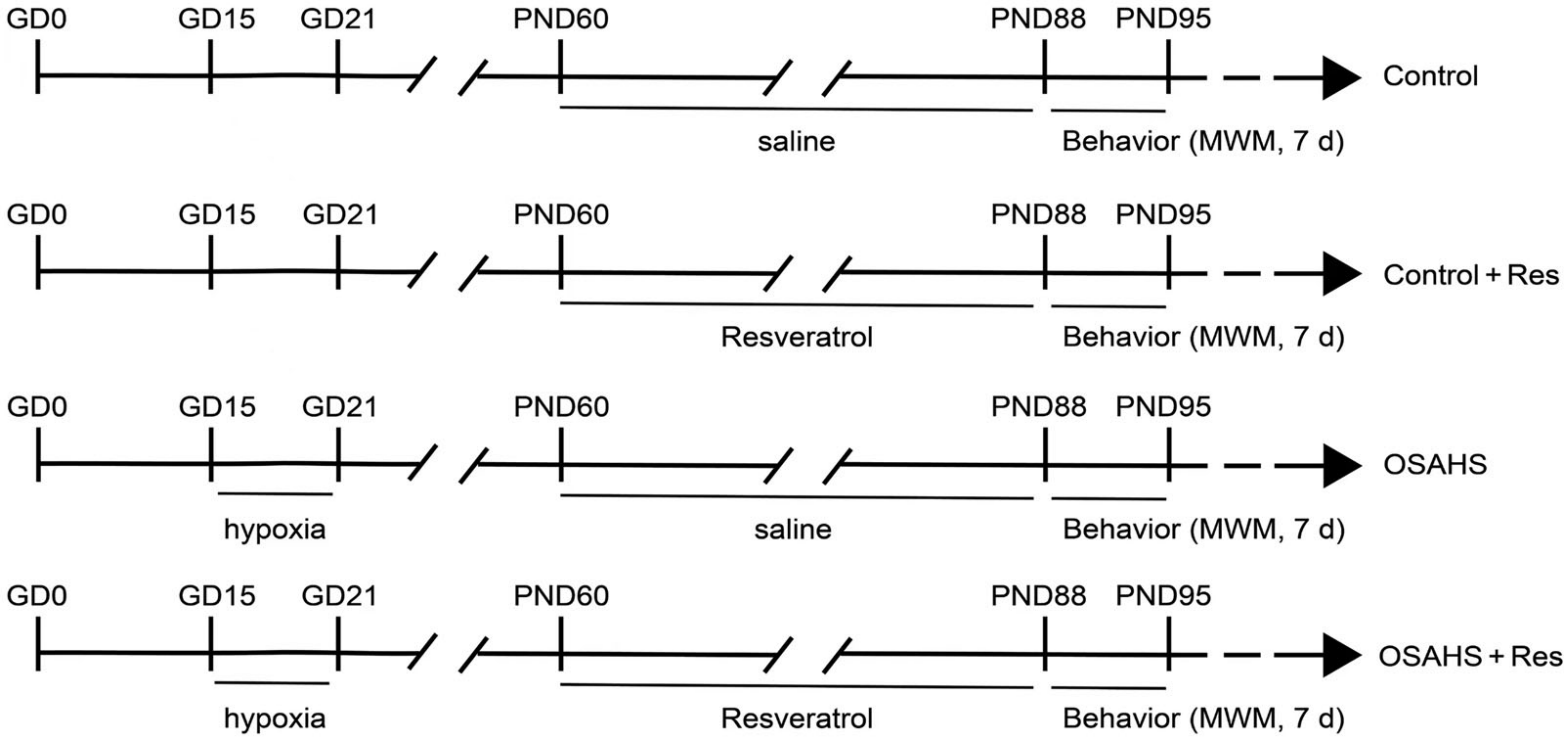
The experimental schedule. GD marks gestational days; PND, postnatal days. Resveratrol is abbreviated as "Res." The Morris water maze (MWM) is the test for spatial learning and memory, with "d" for days.

Figure 1 displays a schematic representation of the treatment schedule. On the 15th day of gestation, expectant mice in their third trimester were subjected to a cycle of hypoxic conditions over a period of 7 days in succession. Using the ProOx‐100 animal hypoxia system, from 9:00 a.m. to 5:00 p.m. daily, with oxygen levels fluctuating between 21% and 10% over a 90 s cycle. This established a mouse model of offspring exposed to late‐pregnancy intermittent hypoxia, whereas the control group was housed in a normoxic hypoxia control system. The day of delivery was designated as the starting point, referred to as postnatal Day 0 (PND0). At PND21, from each litter, one male mouse was randomly chosen and distributed into the following four experimental groups, each comprising eight individuals, a total of 32 animals were used: control group (Control); control group supplemented with resveratrol (Control + RES); hypoxia‐exposed group (mice born to mothers subjected to hypoxic conditions; OSAHS); and hypoxia‐exposed group supplemented with resveratrol (OSAHS + RES). At 2 months of age, the RES‐supplemented control and hypoxia‐exposed groups were administered daily intraperitoneal injections of RES at a dosage of [40 mg/(kg/day)] over a period of 4 consecutive weeks. This dosage was selected based on prior research suggesting its efficacy in mitigating hippocampal inflammation and cognitive deficits while minimizing potential adverse effects (B. Wang et al. 2018; R. Wang, Wu, et al. 2021). RES was dissolved in DMSO (Absin, Ab9189) and diluted with saline containing 5% Tween 80 (Abbexa, abx082610), creating a solution with 5% DMSO and 5% Tween 80 in saline. Behavioral tests were executed between 1:00 p.m. and 6:00 p.m., as shown in Figure 1. The study was conducted with the approval of the Experimental Animal Committee of Hefei Hospital Affiliated to Anhui Medical University, adhering to the ethical guidelines for animal research.

### 2.3 Behavioral Testing: Morris Water Maze

The Morris water maze (MWM) was utilized to assess the spatial learning and memory of the subjects. The test involved a 150 cm diameter, 30 cm high circular black pool, filled with water at a temperature of approximately 22°C. A 10 cm diameter, 24 cm high black platform was submerged in the pool, surrounded by white curtains with three evenly spaced black cues hanging 1.5 m above the water. A tracking camera was set up to record the trajectory of the mouse's movement within the maze, with the footage being analyzed by ANY‐maze software (Sun et al. 2020). (1) Learning Phase: In the 7‐day learning phase for spatial navigation, the platform was submerged 1 cm deep in a fixed quadrant. Mice were given a 30‐s platform acclimation on the 1st day. They were released from different starting points daily, facing the pool's edge, with 60 s to find the platform. Each mouse underwent four training sessions daily, separated by 15‐min breaks. The average swim distance to the platform was recorded, with shorter distances reflecting improved learning. (2) Memory Phase: Completing the learning phase on Day 7, the platform was removed post the final training session. After a rest, the mouse was placed in the water opposite the platform's former site (target quadrant) and allowed a 60‐s free swim. The time and path spent in the target quadrant were recorded to assess memory retention.

### 2.4 Tissue Preparation

Following the MWM test, the offspring were deeply anesthetized using a 2% solution of pentobarbital sodium. Subsequently, the hippocampal region was extracted from the rodents and preserved at an ultra‐low temperature of −80°C in a refrigeration unit for subsequent analysis via ELISA, WB, and RT‐PCR (Y. Zhang et al. 2023).

### 2.5 ELISA

The levels of IL‐1β, TNF‐α, and IL‐6, which are inflammatory cytokines, in the hippocampus were measured using specific assay kits (JYM0531Mo, JYM0218Mo, JYM0012Mo; from Wuhan Colorful Biological Technology Co. Ltd., Wuhan, China), following the manufacturer's instructions precisely: (1) Measure the OD value of the sample using the ELISA instrument, record the data, and set up a negative control group and a blank control group; (2) Add the processed mouse hippocampal tissue samples to the enzyme‐linked plate, and add the corresponding specific antibodies for incubation; (3) Clean the samples to remove unbound antibodies and enzyme‐linked antibodies; (4) Add the enzyme‐linked reagents and incubate again; (5) Perform the color development reaction; (6) Clean the enzyme‐linked plate with wash buffer; (7) Measure the OD value of each well using an ELISA instrument at the appropriate wavelength; (8) Calculate the concentration of inflammatory cytokines in the sample based on the OD values of the control group and sample wells, as well as the data from the standard curve.

### 2.6 RT‐PCR

Total RNA was extracted from hippocampal samples using TRIzol, then converted to cDNA with the PrimeScript RT kit (Takara, RR047A), eliminating gDNA. qPCR was performed with an initial 95°C denaturation for 1 min, followed by 95°C for 20 s and 60°C for 1 min cycles. Gene expression was quantified using the 2‐∆∆Ct method, which is a standard approach for normalizing the expression levels against endogenous controls. The specific primer sequences utilized in the qPCR reactions are detailed in Table 1, ensuring the accuracy and specificity of the gene expression analysis.

**TABLE 1:** Primers used for reverse transcription polymerase chain reaction (RT‐PCR).

Gene	Amplicon size (bp)	Forward primer (5′ → 3′)	Reverse primer (5′ → 3′)
GAPDH	169	GCAGTGGCAAAGTGGAGATTG	CGCTCCTGGAAGATGGTGAT
Arc	121	CCTACAGAGCCAGGAGAATG	CAGCTTCAGGAGAAGAGAGG
GAP‐43	150	GACCAAGAACATGCCTGAAC	AGGGCTCATAGGTAGGAGAG
PSD‐95	110	GCTCCCTGGAGAATGTGCTA	TGAGAAGCACTCCGTGAACT
SIRT1	116	TAATGTGAGGAGTCAGCACC	GCCTGTTTGGACATTACCAC
SYN	124	GCCTACCTTCTCCACCCTTT	GCACTACCAACGTCACAGAC
HIF‐1α	158	TGGACTTGTCTCTTTCTCCG	CGACGTTCAGAACTCATCCT

### 2.7 Western Blotting

Hippocampal tissues were homogenized in RIPA buffer, centrifuged at 12,000 rpm for 15 min to obtain the supernatant. This was mixed with a 5× SDS‐PAGE loading buffer at a 1:4 ratio and heated at boiling point for 15 min. The proteins were separated by SDS‐PAGE at 80 V for about an hour. The resolved proteins were then transferred to a PVDF membrane, which underwent rinsing, blocking, and primary antibody incubation. The antibodies used included those specific for SYP (1:1000; bs‐8845R, BIOSS), PSD‐95 (1:2000; ab238135, Abcam), GAP‐43 (1:5000; ab16053, Abcam), Arc (1:1000; bs‐0385R, BIOSS), and HIF‐1α (1:1000; ab179483, Abcam), as well as a mouse monoclonal antibody against SIRT1 (1:500; sc‐74504, Santa Cruz), with all incubations being performed at 4°C. The protein bands were then analyzed for their intensity using ImageJ software (Media Cybernetics, USA) to perform densitometry, a method that quantifies the optical density of the bands on the membrane.

### 2.8 Statistical Analysis

Data analysis was performed using GraphPad Prism 8.0. Two‐way ANOVA with Tukey's post‐hoc test identified differences in cytokine and synaptic markers. MWM test results were evaluated with repeated measures ANOVA and Tukey's analysis, with a *p *< 0.05 indicating significance.

## 3 Results

### 3.1 RES Ameliorated Cognitive Impairments Precipitated by Prenatal Hypoxia

RES's impact on cognitive deficits, specifically those affecting learning and memory caused by hypoxic exposure, was evaluated on PND90 through the MWM test.

**FIGURE 2:**
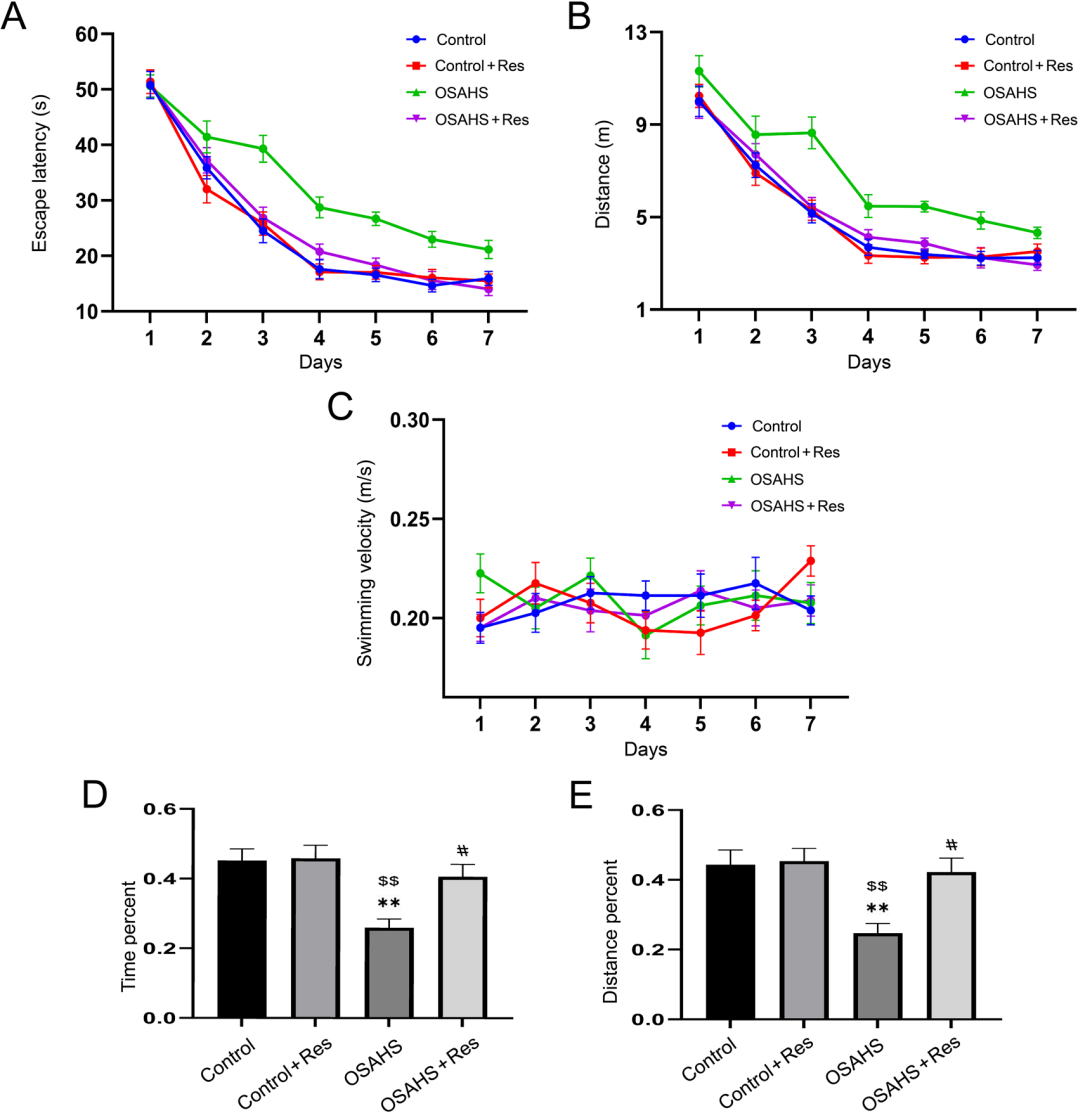
Resveratrol's effect on spatial learning and memory was assessed in offspring mice post maternal hypoxia using the Morris water maze (MWM). (A) Escape latency over 7 days measures the time to find the platform. (B) Total swim distance reflects search effort during training. (C) Swim velocity checks for physical influence on performance. (D) Target quadrant time in the probe test indicates memory of the platform's location. (E) Distance in the target quadrant further measures spatial memory. Significant differences are marked as **p < 0.01 versus Control, $*p* < 0.01 versus Control + Res, #*p* < 0.05 versus OSAHS.

During the learning period, the repeated measures ANOVA indicated substantial effects attributed to the treatment and the passage of time on the escape latency and the distance traveled by the animals (escape latency: *F*(3,28) = 41.28, *p* < 0.01; distance: *F*(3,28) = 25.94, *p* < 0.01; Figure 2A,B). The subsequent post‐hoc analysis disclosed that the rats in the OSAHS group experienced a notably extended escape latency and covered a greater distance compared to their counterparts in the control group. Meanwhile, the swimming velocities across the four groups were found to be statistically indistinguishable from one another (Figure 2C).

In the memory phase, significant variances were observed among the four groups regarding the time and distance spent in the target quadrant (time percentage: *F*(3,28) = 7.851, *p* < 0.01; distance percentage: *F*(3,28) = 7.000, *p* < 0.01; Figure 2D,E). A deeper investigation demonstrated that the OSAHS group had reduced time and distance percentages in the target quadrant when juxtaposed with the control or RES‐only groups. The administration of RES was observed to ameliorate the detrimental impacts caused by hypoxic conditions while it had no significant effect on the Control + RES group. To sum up, these findings suggest that RES supplementation can enhance learning and memory performance that was compromised due to prenatal hypoxia exposure.

### 3.2 RES Mitigated the Inflammatory Cytokine Levels in the Hippocampi of Hypoxia‐Exposed Offspring

**FIGURE 3:**
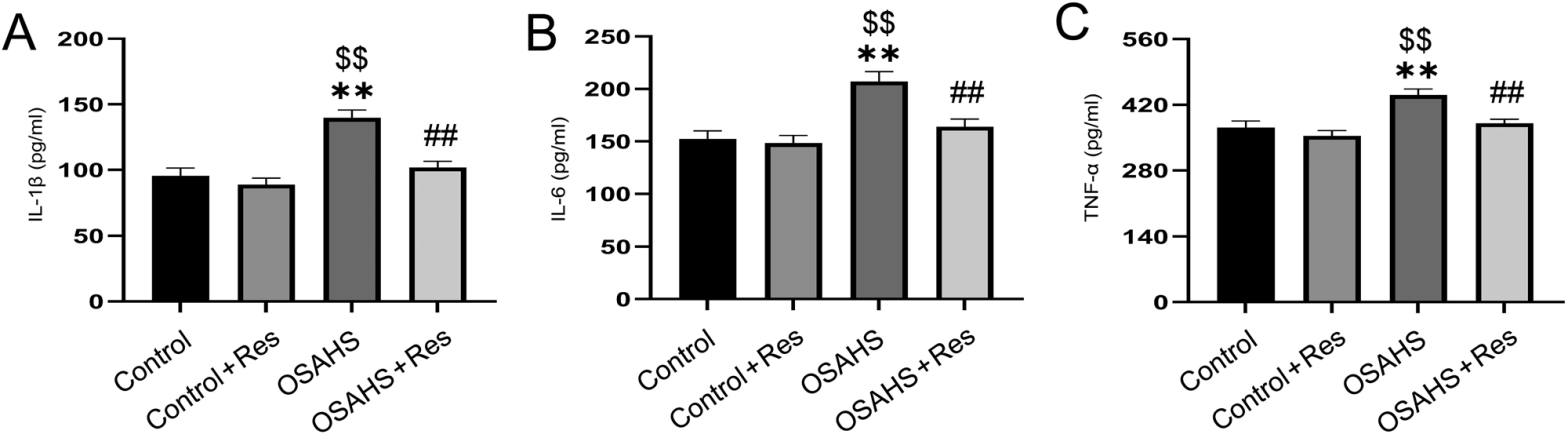
Resveratrol's impact on inflammatory cytokines IL‐1β, IL‐6, and TNF‐α in the hippocampus of gestationally hypoxic mice is depicted. (A) IL‐1β levels are shown. (B) IL‐6 levels are presented. (C) TNF‐α levels are indicated. Statistical significance is denoted as *p < 0.05, **p < 0.01 versus Control; $$*p* < 0.01 versus Res + Control; ##*p* < 0.01 versus OSAHS group.

In assessing the capacity of RES to ameliorate the inflammatory reaction in mice subjected to prenatal hypoxia, we quantified the concentrations of key pro‐inflammatory cytokines, namely IL‐1β, IL‐6, and TNF‐α, within the hippocampal region. The statistical analysis revealed significant differences (IL‐1β: *F*(3,28) = 18.02, *p* < 0.01; IL‐6: *F*(3,28) = 11.55, *p* < 0.01; TNF‐α: *F*(3,28) = 10.30, *p* < 0.01; Figure 3A–C). Post‐hoc tests indicated that exposure to maternal hypoxia significantly elevated the cytokine levels in the offspring's hippocampus (*p *< 0.05), an effect that was attenuated by RES treatment (*p* < 0.05). No significant effect was observed in the Control + RES group. The findings highlight RES's potent effect in markedly reducing the inflammatory reaction within the hippocampal region of offspring mice subjected to maternal hypoxia.

### 3.3 RES Decreased the Levels of SIRT1, HIF‐1α, Arc, GAP‐43, PSD‐95, and SYP Induced by Prenatally Intermittent Hypoxic Exposure

**FIGURE 4:**
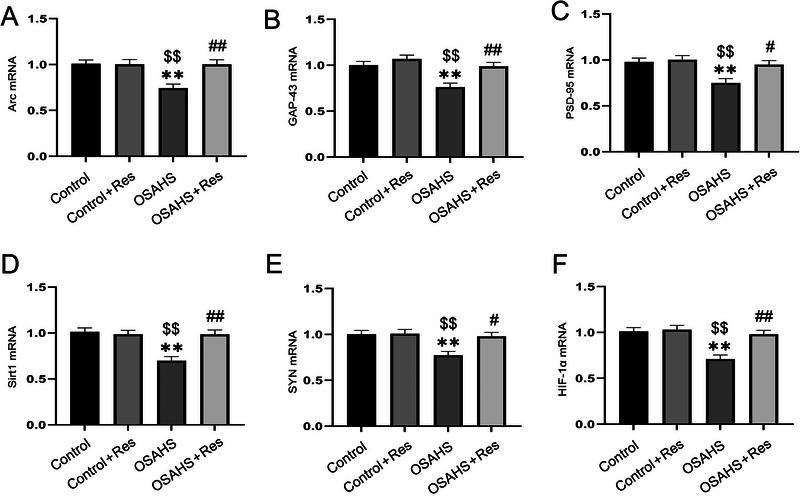
Effects of resveratrol (Res) on Arc, GAP‐43, PSD‐95, Sirt1, SYN, and HIF‐1α in the hippocampus of offspring mice of mothers exposed to hypoxia during pregnancy (A–F).**p < 0.01 compared to the control group; $$*p* < 0.01 compared to the Control + Res group; #*p* < 0.05, ##*p* < 0.01, compared to the OSAHS group.

**FIGURE 5:**
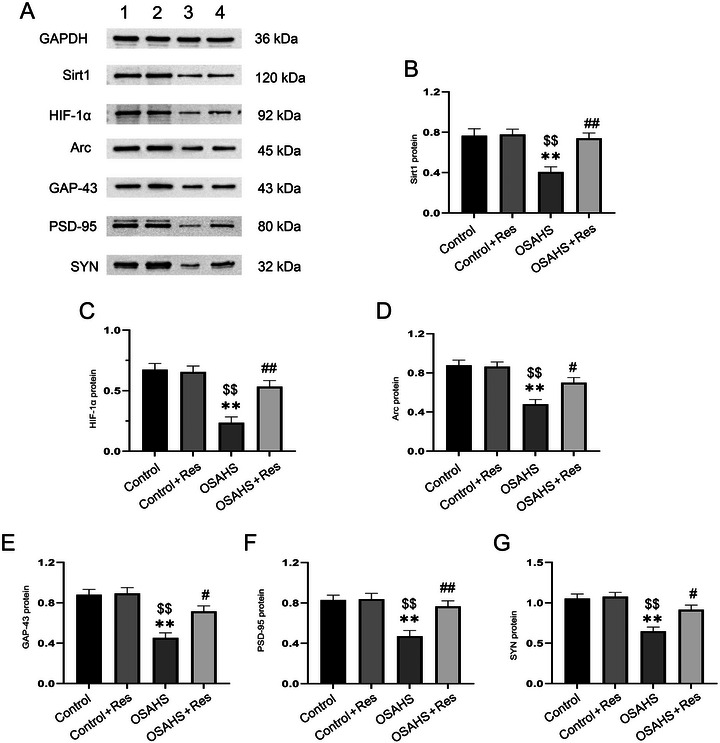
Resveratrol's impact on protein expression in the hippocampus of mice exposed to in utero hypoxia is shown. Western blot bands represent: (1) Control, (2) Control + resveratrol, (3) OSAHS, (4) OSAHS + resveratrol. Quantitative analysis for SIRT1, HIF‐1α, Arc, GAP‐43, PSD‐95, and SYN levels is provided, with significant differences indicated as: **p < 0.01 versus Control; $*p* < 0.01 versus Control + Res; #*p* < 0.05, ##*p* < 0.01 versus OSAHS.

Our research delved into the influence of prenatal hypoxia and subsequent RES administration on the hippocampal expression of several proteins and genes in offspring mice, including SIRT1, HIF‐1α, Arc, GAP‐43, PSD‐95, and SYP. One‐way ANOVA revealed that hypoxic conditions significantly altered the levels of these hippocampal markers (mRNA levels: Sirt1 *F*(3,28) = 11.14, *p* < 0.01; HIF‐1α *F*(3,28) = 6.738, *p* < 0.01;Arc *F*(3,28) = 7.891, *p* < 0.01; GAP‐43 *F*(3,28) = 10.05, *p* < 0.01; PSD‐95 *F*(3,28) = 6.493, *p* < 0.01; SYN *F*(3,28) = 6.738, p < 0.01; Figure 4A–F; protein levels: Sirt1 *F*(3,20) = 9.990, *p* < 0.01; HIF‐1α *F*(3,20) = 16.54, *p* < 0.01; Arc *F*(3,20) = 13.92, *p* < 0.01; GAP *F*(3,20) = 14.77, *p* < 0.01; PSD‐95 *F*(3,20) = 10.29, *p* < 0.01; SYN *F*(3,20) = 13.16, *p* < 0.01, Figure 5A–G). Post‐hoc analyses revealed that OSAHS‐induced cognitive deficits correlated with lowered mRNA and protein levels of key markers (*p *< 0.05), which were alleviated by RES treatment (*p* < 0.05). There was no significant difference observed in the Control + RES group. Essentially, RES appears to neutralize hypoxia's detrimental impact on hippocampal factor expression.

## 4 Discussion

Existing literature indicates that RES activates Sirt1 (Cheng et al. 2016; Wojnarova et al. 2015) and reduces HIF‐1α expression in pancreatic cancer models (Srivani et al. 2020), Sirt1 has a dual role in HIF‐1α regulation: it promotes HIF‐1α stability while potentially inhibiting its transcriptional activity (Joo et al. 2015). This duality may result in varying conclusions regarding the Sirt1‐HIF‐1α interaction, depending on experimental conditions. In our current research we discovered that exposure to hypoxia during the later stages of pregnancy can result in offspring mice exhibiting marked deficits in learning and memory capabilities. This impairment appears to be concurrent with the activation of neuroinflammatory processes and a disruption in synaptic function, potentially linked to the interference with the SIRT1/HIF‐1α signaling cascade. RES has a dual effect; it acts as an antioxidant at low doses but may turn into a pro‐oxidant at high doses (Shaito et al. 2020). The dose‐dependent response of RES leads to varying health effects; while it is considered safe and beneficial at low doses, high doses can cause harmful effects, especially with long‐term use or in combination with other medications. In addition, some clinical trials have reported side effects from RES, such as gastrointestinal discomfort and alterations in blood parameters. In our research, the administration of RES has demonstrated the potential to ameliorate these adverse outcomes by enhancing the expression levels of SIRT1.

### 4.1 RES Ameliorates Cognitive Deficits, Specifically Those Pertaining to Learning and Memory, Which Arise From Exposure to Hypoxic Conditions During the Maternal Period

Throughout the perinatal phase in mammals, encompassing both humans and mice, development is at its most vulnerable to perturbations from external environmental elements. This period is pivotal for growth and can be significantly influenced by the conditions present in the surrounding environment (Gyllenhammer et al. 2022). Intrauterine hypoxia is a common intrauterine condition that can occur in various inflammatory diseases, such as high‐altitude pregnancy, preeclampsia, placental insufficiency, gestational diabetes, maternal obesity, sleep apnea, and others (Mao et al. 2021). PIH deficiency often results in significant alterations in the developmental progress and behavioral characteristics (Vasilev et al. 2016). Studies have shown that rat offspring exposed to PIH on Day 14 of pregnancy exhibit cognitive dysfunction in water maze tests (B. Wei et al. 2016). In our investigation, it was observed that during the acquisition phase of the MWM test, C57BL/6J progeny subjected to hypoxic conditions exhibited an extended duration and greater swimming path to identify the submerged platform. In the retention phase, the offspring from the OSAHS group demonstrated a diminished presence in the target sector and a reduced proportion of the swimming trajectory within that area, signifying cognitive deficits attributed to prenatal oxygen deprivation. The findings from our study suggest that RES significantly ameliorated the cognitive deficits in the progeny caused by maternal hypoxia, as evidenced by a decreased escape latency and a curtailed swimming distance in the learning period of the MWM. In addition, in the memory phase, the OSAHS group supplemented with resveratrol (OSAHS + RES) displayed an enhanced duration and an elevated percentage of swimming distance in the target quadrant, in contrast to the OSAHS group alone.

### 4.2 RES Ameliorates Inflammation Caused by Maternal Hypoxia Exposure

Previous research explored the link between prenatal inflammation and cognitive decline, noting a potential correlation with synaptic protein levels within the hippocampus (Z. Zhang et al. 2022). Cytokines, serving as inflammatory mediators, are produced by a variety of central nervous system cells such as microglia, astrocytes, and neurons. Prominent among these are IL‐1β, IL‐6, and TNF‐α, which are key initiators and are integral to the CNS's normal developmental processes and its reaction to injury (Henrot 2001). Our findings in the OSAHS group revealed increased hippocampal levels of these cytokines compared to controls, indicating that maternal hypoxia might lead to heightened inflammatory responses, thereby affecting cognitive function. RES has demonstrated the ability to improve cognitive function in aging rats by inhibiting the production of pro‐inflammatory cytokines (Gocmez et al. 2016). In our research, we noted a decrease in inflammatory markers in the group treated with resveratrol (OSAHS + RES) compared to the untreated OSAHS group. RES appeared to neutralize the increase in pro‐inflammatory cytokines caused by prenatal hypoxia, suggesting a positive effect on the inflammatory processes initiated by PIH.

### 4.3 RES Mitigates the Impairment of Synaptic Function That Arises From Exposure to Hypoxic Conditions During the Maternal Period

The hippocampus, a critical brain region instrumental in the processes of memory formation and retrieval, particularly excels in spatial learning and memory tasks. Within this structure, synaptic plasticity stands as a fundamental biological process that facilitates learning and memory mechanisms (Magee and Grienberger 2020). Research indicates that mice subjected to prenatal hypoxia demonstrate pronounced impairments in spatial learning and memory, characterized by a decrease in synaptic density, heightened tau protein phosphorylation, and increased astrocyte and microglial cell activation, alongside diminished expression of hypoxia‐inducible factors (X. Zhang et al. 2013). The activation of SIRT1 is known to bolster synaptic plasticity, while its functional impairment can lead to the opposite effect (Gao et al. 2010). Evidence suggests that mice with a targeted knockout of brain‐specific SIRT1 exhibit diminished synaptic plasticity (Herskovits and Guarente 2014). SIRT1 is also implicated in the modulation of synaptic plasticity and memory consolidation, potentially through miRNA‐mediated mechanisms (Choi and Kemper 2013). The Arc protein is essential for synaptic plasticity within the hippocampus, which is vital for the consolidation of memory (LaLumiere et al. 2017). Arc's role in synaptic plasticity involves the trafficking of AMPA‐type glutamate receptors (AMPARs) via endocytic processes (Pastuzyn et al. 2018). The function of PSD‐95 is intricately linked to the proper modulation of synaptic plasticity (Parlog et al. 2014). Synaptic proteins such as PSD‐95 and SYN are pivotal for synaptic plasticity and cognitive function (Shi et al. 2020). GAP‐43 associated with growth cones, synaptic plasticity, and synaptic regeneration, is implicated in the regulation of synaptic plasticity in the adult brain, which is crucial for memory storage (Jasperse et al. 2007; C. Wang et al. 2015). Arc and GAP‐43 are fundamental for synaptic plasticity and neuronal development, whereas PSD‐95 and SYP are indispensable for maintaining synaptic structure and function. Our results indicate that offspring from mothers exposed to hypoxia exhibit notably decreased levels of SIRT1, HIF‐1α, SYP, Arc, GAP‐43, and PSD‐95. This decrease implies that synaptic plasticity disruptions could be the root of cognitive impairments linked to PIH, possibly because of the disruption of the SIRT1/HIF‐1α signaling pathway. Furthermore, in alignment with its role as an activator of SIRT1, RES was found to reverse the downregulation of these proteins and ameliorate the learning and memory deficits induced by PIH. Collectively, our outcomes propose that RES may counteract cognitive impairments in rats resulting from maternal hypoxia during pregnancy by modulating the hippocampal SIRT1/HIF‐1α pathway.

#### 4.3.1 Limitation

One limitation of our study was the exclusive reliance on the MWM for evaluating spatial learning and memory behaviors, omitting other hippocampal cognitive tests like the Y‐maze and Novel Object Recognition. In addition, we exclusively examined the impact of maternal hypoxia on male offspring, without assessing the potential for gender‐specific outcomes that could arise from maternal exposure to hypoxic conditions. Third, the study's reliance on a single dosage and administration period of RES may not reflect the optimal therapeutic window or dosage required for maximal efficacy, future studies should consider including female offspring and exploring a range of dosages and treatment durations to provide a more comprehensive understanding of RES's neuroprotective effects. Furthermore, we did not incorporate the use of immunohistochemical methods to scrutinize the expression patterns of proteins associated with synaptic function, such as Arc, GAP‐43, PSD‐95, and SYP, across various subregions of the hippocampus. Future research may increase the application of microscopy techniques for electrophysiology, synaptic protein expression, and immunofluorescence.

## 5 Conclusion

Our research indicates that PIH exposure could provoke inflammation and synaptic dysfunction, with RES potentially reversing cognitive deficits by influencing the SIRT1/HIF‐1α pathway. RES's intervention is suggested to regulate inflammatory and synaptic markers in the hippocampus, reducing pro‐inflammatory cytokines and increasing Arc, GAP‐43, PSD‐95, and SYP levels. This suggests RES's therapeutic potential for cognitive impairments due to prenatal hypoxia. Further studies are needed to elucidate these mechanisms.

## Author Contributions

**Xun He**: conceptualization, writing–original draft. **Ziwei Cao**: conceptualization, writing–original draft. **Xinyi Chen**: methodology, validation. **Jie Hu**: methodology, validation. **Jiaxuan Li**: software, formal analysis. **Xinhui Jia**: software, formal analysis. **Juncang Wu**: writing–review and editing, visualization. **Xuechun Liu**: resources, data curation, funding acquisition, project administration.

## Ethics Statement

All procedures complied with the ethical standards of the Experimental Animal Committee at Hefei Hospital Affiliated Anhui Medical University, and were approved with the reference (No. 2024‐scientific research‐087).

## Conflicts of Interest

The authors declare no conflicts of interest.

### Peer Review

The peer review history for this article is available at https://publons.com/publon/10.1002/brb3.70413

## Data Availability

The authors pledge to supply the underlying data that substantiates the findings of this paper, ensuring no unwarranted withholding.
